# Neuroanatomy and Neurophysiology of the Corrupt-Prone Brain: Where Morality, Cognition, and Behaviour Intersect

**DOI:** 10.21315/mjms-04-2026-s02

**Published:** 2026-06-30

**Authors:** Jafri Malin Abdullah, Geshina Ayu Mat Saat, Faiz Mustafar, Syaheerah Lebai Lutfi

**Affiliations:** 1 Department of Neurosciences, School of Medical Sciences, Universiti Sains Malaysia, Health Campus, 16150 Kubang Kerian, Kelantan, Malaysia; 2 Brain and Behaviour Cluster, Hospital Pakar Universiti Sains Malaysia, Universiti Sains Malaysia, Health Campus, 16150 Kubang Kerian, Kelantan, Malaysia; 3 Department of Neurosciences, Hospital Pakar Universiti Sains Malaysia, Jalan Raja Perempuan Zainab 2, 16150 Kubang Kerian, Kelantan, Malaysia; 4 Forensic Science Programme, School of Health Sciences, Universiti Sains Malaysia, Health Campus, 16150 Kubang Kerian, Kelantan, Malaysia; 5 School of Computer Sciences, Universiti Sains Malaysia, 11800 USM, Penang, Malaysia; 6 College of Medicine and Health Sciences, Sultan Qaboos University, Seeb, Sultanate of Oman

**Keywords:** corruption, bribery, brain, neuroimaging, neuropsychology, cognition, mind

## Abstract

In Malaysia, corruption analyses and brain, and mind and neuroscience; are two critical domains of enquiry that seek to advance research that bridges medicine, clinical neurophysiology and neurotechnology by 2050. Local universities, both public and private, must initiate studies into how corruption and malpractice—and their resulting economic losses to the nation—can be prevented. An understanding of neuroanatomy, neurophysiology. and neurochemistry will hopefully advance knowledge of how the human brain can be trained to resist corruption and, eventually, be rehabilitated.

## Introduction

Corruption is traditionally examined through legal, political and socio-economic frameworks. While these perspectives remain essential, they do not fully explain why individuals differ markedly in their vulnerability to and continuous involvement in corrupt behaviour under similar circumstances. Over the past several decades, advances in cognitive neuroscience have provided compelling evidence that moral reasoning, honesty, and ethical decision making are rooted in identifiable brain structures and neurophysiological processes (1). This editorial highlights emerging neuroscientific insights into corruption, with specific emphasis on the neuroanatomical and neurophysiological substrates underlying moral and immoral behaviour.

National legal frameworks vary in their definitions of corruption and in the classification of behaviours deemed to constitute statutory violations. Within these frameworks, corruption is generally conceptualised as the abuse of entrusted authority for personal or private gain. From a neuroscientific perspective, this behavioural outcome reflects complex interactions between cognitive control, emotional regulation, reward processing, and social cognition. These functions are mediated by distributed neural networks, particularly within the frontal lobe, and are modulated by developmental, genetic, and cultural factors (2–12). The intersection of corruption thinking and brain imaging is an emerging area of neuroeconomics and political neuroscience that uses functional magnetic resonance imaging (fMRI) and functional near-infrared spectroscopy to map how the human brain calculates the gains, moral trade-offs, and social risks of corrupt behaviour. Neuroimaging reveals that the decision to engage in corruption is not a simple choice but a complex, multilayered cognitive process in which localised brain regions weigh personal financial rewards against the psychological moral cost of dishonesty (13). Corruption rarely happens in isolation; it usually requires a corrupt alliance (e.g. a briber and a bribe taker). Advanced multi-brain imaging (hyperscanning) tracking pairs of individuals has uncovered the following:

- Honesty Norms: Brain-to-brain synchronisation within the right dorsolateral prefrontal cortex (DLPFC) between two people correlates with fewer corrupt behaviours. Higher synchronisation in this region reinforces shared moral judgement and pushes individuals towards honest actions.
- Reciprocity Norms: Conversely, neural synchronisation in the right temporoparietal junction (rTPJ) can actually drive greater corrupt collaboration. In these scenarios, misplaced empathy and a desire to cooperate with one’s partner override broader social norms of honesty.

As corruption is multi-layered, these neurobiological foundations are significantly modulated by sociocultural variables; most existing frameworks lack cross-cultural validation and often overlook the influence of indigenous ethical landscapes on brain activity (14).

## Neuroanatomy and the Neurodevelopmental Basis of Moral Behaviour

The adult human brain is organised into specialised regions responsible for motor control, sensory processing, cognition and behaviour (Figures 1 and 2). Among these, the prefrontal cortex (PFC) plays a pivotal role in higher-order cognitive functions, including judgement, impulse inhibition, and moral reasoning. Importantly, the PFC undergoes prolonged development from childhood through early adulthood, a process characterised by synaptic pruning, myelination, and refinement of neural connectivity.

This extended developmental trajectory parallels the maturation of moral judgement, informed decision making, and behavioural self-regulation. Neurodevelopmental studies demonstrate that both prosocial and antisocial behavioural tendencies are shaped during this period by environmental exposure, cultural norms, media influence, and social learning. Consequently, moral behaviour should be viewed as a dynamic construct rather than a fixed trait (2–12).

## Functional Neuroanatomy of Moral Cognition

Within the PFC, functionally distinct subregions contribute to different aspects of moral cognition. The medial PFC is primarily involved in value-based decision making and self-referential processing. The orbitofrontal cortex evaluates reward and punishment, integrating emotional and motivational signals. The lateral PFC supports planning, reasoning and cognitive control (8).

The ventromedial PFC is most consistently linked to both moral and immoral behaviour, with the DLPFC and orbitofrontal cortex playing complementary roles in regulating, overriding or rationalising such behaviours (9, 10).

Among these regions, the DLPFC has been consistently implicated in honest behaviour and resistance to unethical conduct. Experimental neuroimaging studies indicate that DLPFC activation increases when individuals subordinate self-interest to moral norms. Conversely, reduced DLPFC activity is associated with greater susceptibility to bribery and corrupt decision making (4, 7).

The temporoparietal junction complements the DLPFC by encoding concern for others’ welfare and social reciprocity. While reciprocity is a fundamental social mechanism, its excessive prioritisation over cognitive control may facilitate corrupt collaboration. Thus, corruption may arise not from a single brain region but from an imbalance within a distributed moral network (8–11).

## Neurophysiological and Neurochemical Modulation

Moral behaviour is further shaped by neurophysiological processes, particularly neurotransmitter systems. Serotonin, dopamine, noradrenaline, gamma-aminobutyric acid (GABA), glutamate, and acetylcholine modulate attention, emotional regulation, reward sensitivity, and impulse control. Variations in these systems influence individual differences in ethical decision making (12, 13).

Dopaminergic signalling within prefrontal and mesolimbic pathways encodes subjective value and reward anticipation, both of which are central to corrupt behaviour. Cross-cultural neuroimaging studies suggest that dopamine receptor gene expression and neurotransmitter balance are associated with structural and functional differences in the PFC, respectively. Similarly, GABAergic modulation within the DLPFC predicts working memory capacity, and cognitive control; which are essential for suppressing unethical impulses.

Notably, experimental neuromodulation studies have demonstrated that targeted alteration of DLPFC activity can influence resistance to bribery. These findings provide causal evidence that moral behaviour is not merely a theoretical or philosophical construct but is biologically grounded and experimentally modifiable (6–13, 15).

## Cognitive Pathways of Corrupt Behaviour

Corruption most commonly occurs in organisational contexts, including those involving business transactions and power dynamics. Research shows that institutional narratives and framing strategies play a critical role in shaping how individuals cognitively process corruption (Table 1). Studies demonstrate that accountability statements often employ reformist, deferral, or minimisation frames; which convey ethical leniency and reduce perceptions of blame. These communicative mechanisms act as moral disengagement cues, allowing individuals to rationalise misconduct and sustain corrupt practices within organisations. From a criminological perspective, such framing illustrates how corruption is embedded in organisational discourse, enabling individuals to cognitively distance themselves from responsibility while maintaining an appearance of legitimacy.

Psychological and criminological assessment studies reveal that corrupt behaviour is often evaluated through surveys, scenarios, and bribery games; each exposing how individuals cognitively process opportunities for misconduct and rationalise unethical choices. These instruments highlight the variability of cognitive responses depending on context, theoretical framing, and sample characteristics; reinforcing the view that corruption is not random but structured by decision-making processes and evaluative mechanisms.

Complementing these assessments, dual-process social psychological models demonstrate that ideological orientations and worldviews influence corrupt intentions and attitudes toward corrupt actors. Grounded theory research with convicted offenders provides direct insight into the decision-making process underlying corrupt behaviour, which is shaped by contextual pressures and personal justifications.

Empirical evidence also highlights the role of moral disengagement and cognitive vulnerabilities in sustaining corruption. Survey data from Latin America reveal that tolerance for bribery is strongly linked to weakened moral identity and disengagement tendencies (19). Similarly, studies of public servants in Brazil show that low self-control and weak ethical cultures foster corrupt behaviour, while ethical leadership and risk perception strengthen organisational integrity (20).

These findings underscore that corruption is not simply opportunistic but emerges from cognitive pathways shaped by individual traits and organisational environments. Criminology thus situates corruption within the interplay of psychological predispositions, cognitive pathways, and structural conditions; explaining how individuals justify misconduct through disengagement and rationalisation in organisational and societal contexts.

## Ethical and Societal Implications

Neuroscientific insights into corruption raise important ethical considerations. Current evidence does not support the use of neuroimaging or neurochemical profiling to diagnose or predict corrupt behaviour in individuals. Rather, these findings should be interpreted as population-level insights that inform prevention strategies, education, and institutional design.

Social media research increasingly demonstrates how digital platforms shape and amplify unethical ideologies and practices. Reports circulated online by governments and individuals alike reveal the material rewards and lucrative incentives derived from corrupt activities (26–29). Yet, both the mechanisms of moral inhibition and the deterrent force of national punitive sanctions appear insufficient when confronted with the relentless stream of media coverage exposing organisational misconduct, corruption, and abuse of power (27–30).[Table t2-01mjms3303_ed]

Neuroscientific findings further reveal that corrupt collaboration is sustained through braintobrain synchronisation. Equally important are the collective and institutional dimensions of corruption, in which moral disengagement and narrative framing shape societal tolerance. Evidence shows that corruption is reinforced when organisational cultures permit disengagement from ethical standards, particularly in environments with weak selfcontrol and absent ethical leadership. At the societal level, institutional narratives can obscure accountability by reframing misconduct in ways that minimise blame or externalise responsibility, conveying ethical leniency to wider audiences (31–35).

A further ethical concern lies in the potential for corruption to reshape social norms in negative ways. Studies in social psychology show that when corruption becomes widespread, societies adjust moral boundaries to accommodate misconduct, gradually normalising unethical behaviour as acceptable practice. This shift erodes collective resistance, weakens trust in institutions, and embeds corruption into everyday interactions. Such shifts in norms are particularly damaging because they perpetuate cycles of misconduct, making reform efforts more difficult and embedding corruption within organisational and societal cultures.

Understanding the neural basis of moral behaviour underscores the importance of environments that strengthen cognitive control, ethical reasoning, and emotional regulation. Educational systems, organisational structures, and public policies that collectively promote transparency and accountability may indirectly support the neural mechanisms underlying honest behaviour.

## Implications for Organisational Governance

Emerging evidence in social psychology and organisational behaviour demonstrates that corruption within organisations is sustained not only by individual choices but also by collective cognitive processes (Table 3). Multibrain studies reveal that corrupt collaboration synchronises neural activity in decisionmaking regions, indicating that unethical behaviour can be reinforced through shared cognitive engagement rather than through isolated individual acts. Related work shows that exposure to corruption-related messaging alters perceived closeness to political actors and authority figures, thereby reshaping attitudes towards legitimacy and weakening resistance to misconduct. These findings suggest that organisational environments can foster conditions in which corruption becomes cognitively embedded and socially reinforced (35).

Economic inequality has been shown to intensify corrupt behaviour by fostering perceptions of unfairness and amplifying selfinterest, thereby creating organisational climates in which misconduct is rationalised as necessary for survival or advancement. At the organisational level, moral disengagement mechanisms further erode ethical standards, enabling individuals to rationalise and justify corrupt practices. Institutional narratives also play a critical role, framing corruption in ways that normalise misconduct and diminish accountability. Broader analyses conceptualise corruption tolerance as a moral and social cognition process, showing how societies and organisations adjust moral boundaries to accommodate unethical behaviour, which may become entrenched and normalised over time (31–35).

Behavioural public policy research demonstrates that corruption can be reduced through structural interventions, such as nudges, sludge audits, and digital choice architectures, which limit opportunities for discretionary abuse and weaken cognitive pathways that legitimise misconduct. Complementary evidence from government bureaucracies indicates that embedding anti-corruption values is closely tied to subjective wellbeing and productive behaviour. Environments that promote efficiency and integrity further strengthen the intention to resist corruption.

The role of organisational culture and the ethical climate in shaping governance outcomes must also be considered in this discourse. Evidence shows that weak ethical cultures and low selfcontrol foster moral disengagement, while ethical leadership, risk perception and fair compensation strengthen integrity and reduce tolerance for misconduct (33, 34).

## Conclusion

Corruption-related behaviour arises from complex interactions within distributed brain networks, particularly involving the dorsolateral and ventromedial prefrontal cortices. These networks integrate sensory input, emotional signals, memory and value assessment to guide moral decision making. Neuroscientific evidence suggests that strengthening cognitive control within these circuits enhances honesty, whereas functional suppression increases vulnerability to unethical conduct.

Beyond the neurophysiological and neurochemical modulation of individuals engaged in corrupt behaviour, collective processes within organisations enable corruption to emerge and persist. Governance reforms must integrate economic, behavioural and psychological dimensions, addressing corruption not only as a legal violation but also as a phenomenon sustained by collective cognition, moral disengagement and institutional culture.

While neuroscience cannot replace legal or moral accountability, it provides a valuable complementary framework for understanding corruption as a neurocognitive phenomenon shaped by development, culture and biology. Continued interdisciplinary dialogue is essential to ensure that these insights are applied responsibly for societal benefit.

## Figures and Tables

**Figure 1:**
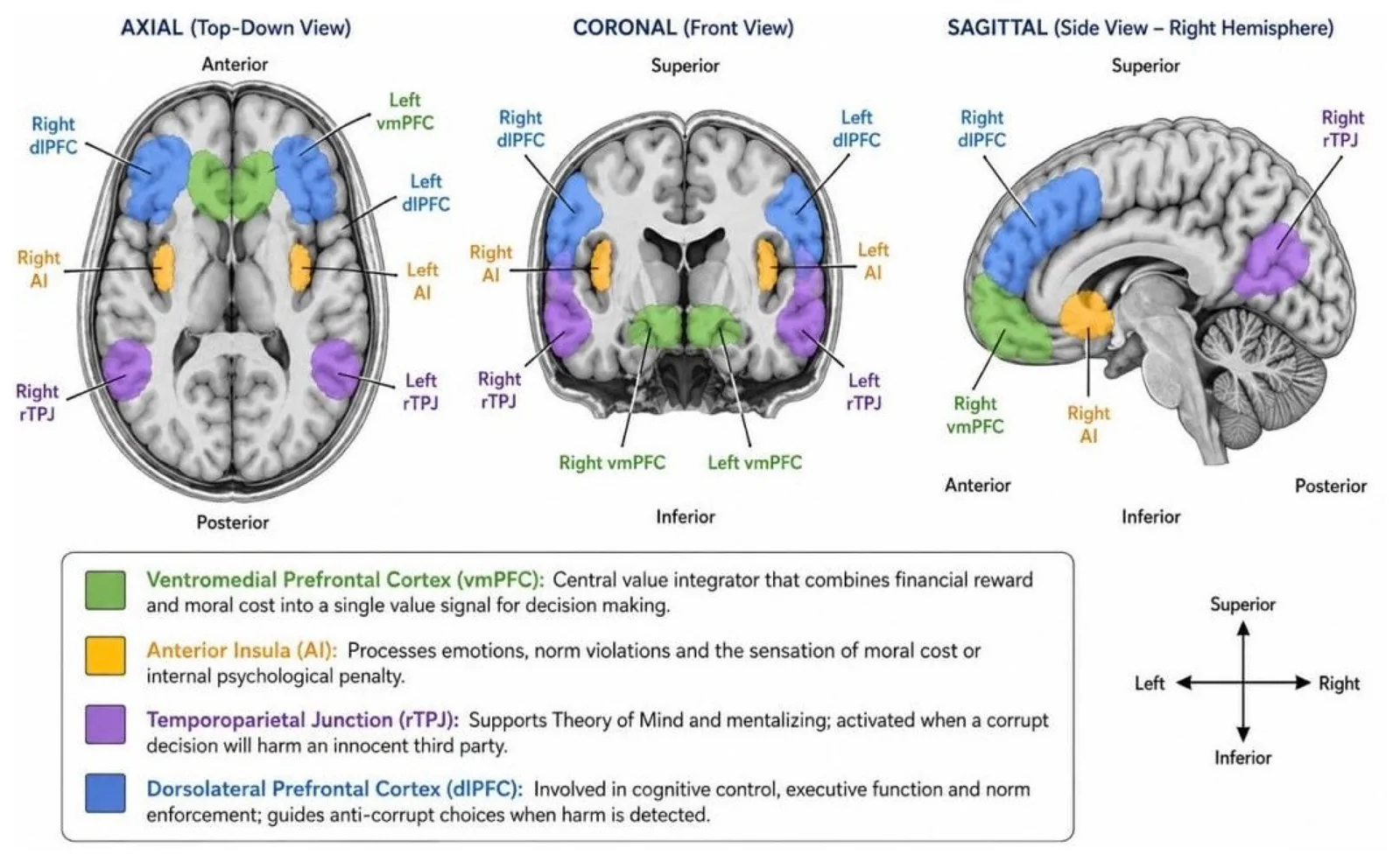
Neural anatomy of a corrupt decision.

**Figure 2:**
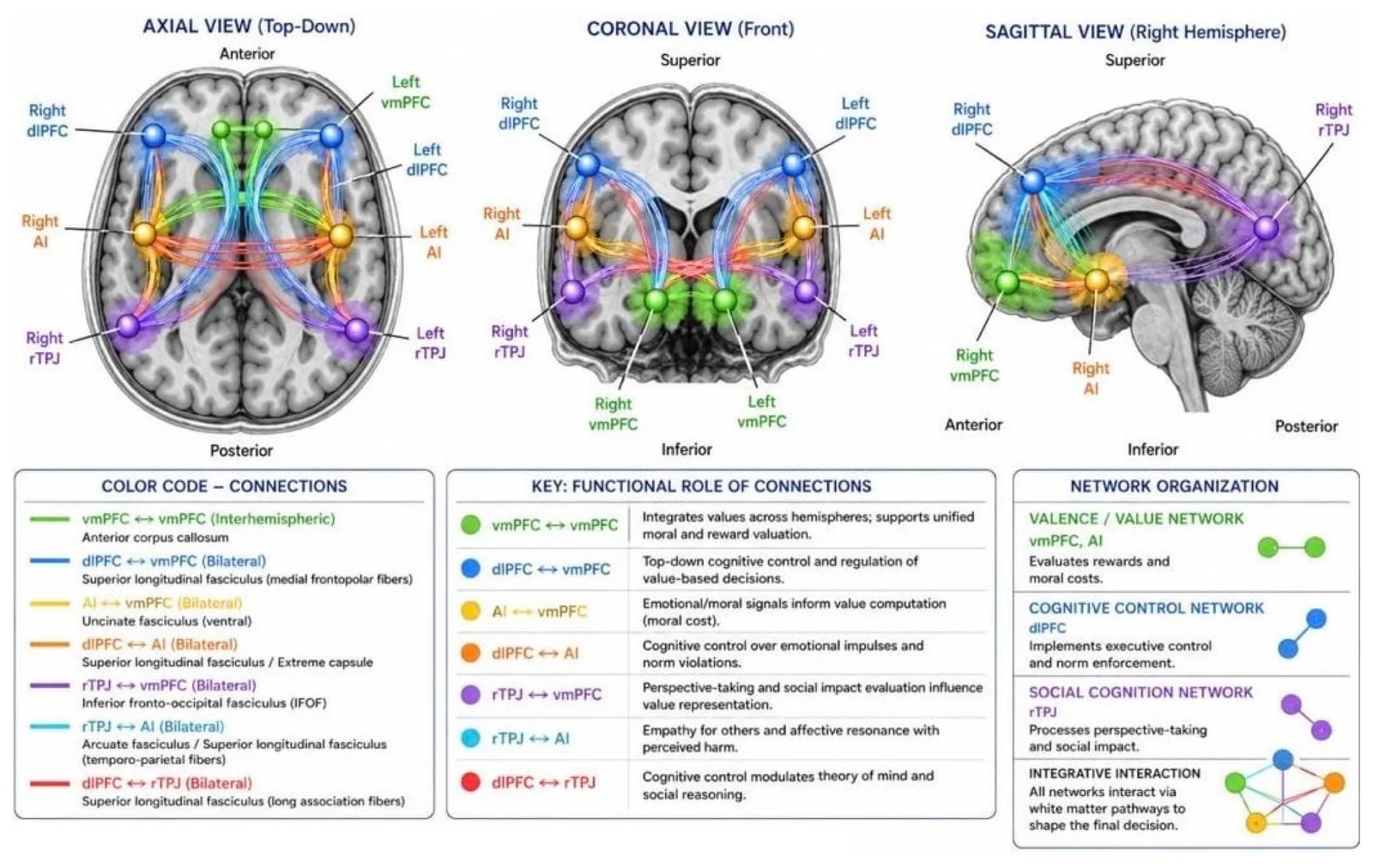
Tractography map showing major white matter pathways linking key brain regions involved in corrupt decision making. The tractography pathways are probabilistic estimates from diffusion MRI and represent major white matter anatomical connections.

**Table 1:** Comparison of cognitive pathways of corrupt behaviour.

Pathway	Core cognitive mechanism	Insight
Psychological assessment pathway (16)	Evaluation of corrupt behaviour through surveys, scenarios and bribery games reveals how individuals cognitively process opportunities for misconduct	Corruption is structured by evaluative mechanisms—how individuals rationalise choices across different organisational settings
Dual process ideological pathway (17)	Ideological orientations and worldview beliefs shape corrupt intention and attitudes toward corrupt actors	Distinct cognitive routes underpin behaviour and perception; ideology and moral disengagement converge to sustain tolerance for corruption
Decision making pathway (18)	Sequential cognitive stages—problem recognition, information search, and evaluation—frame corruption as a rational solution	Corruption is cognitively constructed as viable behaviour; it situates misconduct within structured reasoning shaped by contextual pressures

**Table 2 t2-01mjms3303_ed:** Societal implications of corruption.

Dimension	Key findings	Societal implications
Neural basis of moral judgement (21)	Cognitive conflict in moral dilemmas involves both reasoning and emotional processes, reflecting tension between utilitarian and deontological values	Ethical decision making is shaped by competing neural subsystems, underscoring the complexity of predicting corrupt behaviour
Somatic and emotional markers (22)	Immoral and corrupt behaviour is influenced by somatic markers that bias decision making under uncertainty	Emotional signals can override rational control, raising ethical concerns about vulnerability to corruption in highpressure organisational contexts
Collective neural engagement (5)	Corrupt collaboration is sustained through braintobrain synchronisation in decisionmaking regions	Corruption can become socially reinforced through shared cognition, complicating prevention strategies at the organisational and societal levels and raising the difficult questions about collective accountability
Framing and social cognition (23, 24)	Narrative framing and social norms shape tolerance of corruption, altering perceptions of legitimacy and accountability	Institutional narratives can launder misconduct, embedding corruption into social norms, weakening collective resistance, and ultimately eroding ethical boundaries
Societal norm shifts (25)	Widespread corruption reshapes moral boundaries, normalising misconduct as acceptable practice	Ethical concern arises when corruption alters collective norms, eroding trust in institutions and embedding misconduct into daily life

**Table 3:** Basis for corruption in organisational governance.

Basis	Key findings	Governance implications
Economic inequality	Greater inequality fosters corrupt behaviour by intensifying selfinterest and perceptions of unfairness	Governance must address structural inequalities to reduce environments where corruption is rationalised as survival or advancement
Behavioural public policy	Nudges, sludge audits, and digital choice architectures reduce opportunities for misconduct	Reforms should embed behavioural insights into organisational design, moving beyond punitive regulation toward preventive structures
Anti-corruption values in bureaucracy	Subjective wellbeing and productive behaviour strengthen intentions to uphold anticorruption values	Governance must integrate psychological and economic dimensions, fostering environments that promote integrity and institutional resilience
Organisational culture and ethical climate	Weak ethical cultures and low selfcontrol foster moral disengagement, while ethical leadership and risk perception strengthen integrity	Governance reforms must prioritise ethical leadership and accountability structures to counteract the rationalisation of misconduct and prevent corrupt behaviour from becoming entrenched
